# Differential Expression of Fibrosis-Related Genes in Intrauterine Adhesions and Cesarean Scar Defects: A Cohort Study

**DOI:** 10.3390/jcm15052021

**Published:** 2026-03-06

**Authors:** Loredana Maria Toma, Natalia Simionescu, Raluca Balan, Demetra Socolov, Ioana-Sadiye Scripcariu, Florin Zugun-Eloae, Mihaela Tirnovanu, Daniela Viorelia Matei, Razvan Socolov

**Affiliations:** 1 Department of Biomedical Science, Faculty of Bioengineering, University of Medicine and Pharmacy “Grigore T Popa”, 700454 Iasi, Romania; loredana-toma@umfiasi.ro (L.M.T.); daniela.matei@umfiasi.ro (D.V.M.); 2 “Elena Doamna” Clinical Hospital of Obstetrics and Gynaecology, 700398 Iasi, Romania; raluca.balan@umfiasi.ro (R.B.);; 3 Centre of Advanced Research in Bionanoconjugates and Biopolymers, “Petru Poni” Institute of Macromolecular Chemistry, 700487 Iasi, Romania; 4 Department of Morpho-Functional Sciences I—Histology, University of Medicine and Pharmacy “Grigore T Popa”, 700115 Iasi, Romania; 5 Department of Mother and Child Medicine—Obstetrics and Gynecology, University of Medicine and Pharmacy “Grigore T Popa”, 700115 Iasi, Romania; demetra.socolov@umfiasi.ro (D.S.); ioana.scripcariu@umfiasi.ro (I.-S.S.); mihaela.tirnovanu@umfiasi.ro (M.T.); 6 “Cuza Voda” Clinical Hospital of Obstetrics and Gynaecology, 700038 Iasi, Romania; 7 Department of Morpho-Functional Sciences I—Immunology, University of Medicine and Pharmacy “Grigore T Popa”, 700115 Iasi, Romania; eloae.zugun@umfiasi.ro

**Keywords:** intrauterine adhesions, isthmocele, endometrial fibrosis, TGF-β1, SMAD2, SMAD3, fibronectin, TGF-β1/SMAD signaling, gene expression, qRT-PCR

## Abstract

**Objectives:** This study aimed to characterize the expression patterns and interrelationship of key fibrosis-related markers—*TGF-β1*, *SMAD2*, *SMAD3*, and *fibronectin*—in human endometrial tissue, and to explore their potential diagnostic relevance in differentiating intrauterine adhesions (IUAs) from cesarean scar defects (isthmocele), with a particular focus on underlying fibrotic remodeling processes. **Methods:** Endometrial samples were obtained from women diagnosed with IUAs, isthmocele, or without uterine pathology. Total RNA was extracted from all specimens, and gene expression levels were quantified using real-time quantitative polymerase chain reaction (PCR). Statistical analyses included intergroup comparisons, parametric and non-parametric correlation analysis, multivariable linear and logistic regression models, and receiver operating characteristic (ROC) curve analysis to explore the discriminatory potential of the evaluated markers. **Results:** Significant positive correlations were observed across the study population between *SMAD2* and *SMAD3* (r = 0.892; *p* = 0.001), *SMAD2* and *TGF-β1* (r = 0.697; *p* = 0.001), and *SMAD3* and *TGF-β1* (r = 0.910; *p* = 0.001), indicating coordinated activation of profibrotic signaling pathways. ROC curve analysis showed high discriminatory performance for isthmocele across all evaluated markers, with area under the curve (AUC) values of 0.976 for *SMAD3*, 0.961 for *TGF-β1*, 0.913 for *fibronectin*, and 0.928 for *SMAD2* (all *p* = 0.001). In contrast, although elevated expression levels of fibrotic markers were observed across different American Fertility Society (AFS) stages in IUAs, these differences did not reach statistical significance. **Conclusions:** This study provides molecular evidence distinguishing isthmocele from IUAs with respect to fibrosis-related signaling in human endometrial tissue. The markedly elevated and coordinated expression of *TGF-β1*, *SMAD2*, *SMAD3*, and *fibronectin* in isthmocele reflects activation of post-cesarean fibrotic remodeling pathways. However, given the limited sample size and the exploratory nature of the analyses, larger cohorts and future studies are required to validate these findings and to allow extrapolation of the results to the general population. At this stage, these biomarkers should therefore be regarded as indicators of underlying pathophysiological processes.

## 1. Introduction

Endometrial fibrosis is a pathological response involving excessive extracellular matrix (ECM) deposition and tissue remodeling, contributing to infertility in conditions such as intrauterine adhesions (IUAs) and isthmocele [1,2,3]. While both conditions share fibrotic components, they differ in etiology and clinical context. Intrauterine adhesions (IUAs) are a pathological condition of the uterine cavity characterized by the presence of abnormal adhesions, which may involve the uterine walls and, in some cases, the endocervical canal. The formation of IUAs leads to significant alterations in the endometrial architecture, resulting in menstrual abnormalities, cyclic pelvic pain, uterine cavity obliteration, and, in severe cases, infertility and recurrent miscarriages [1,4]. Intrauterine adhesions often follow surgical trauma (dilation & curettage, hysteroscopy) or infections, resulting in endometrial scarring and glandular atrophy [5]. In contrast, isthmocele is a cesarean scar defect characterized by myometrial discontinuity, chronic inflammation, and fibrosis at the uterine incision site following cesarean section [6,7].

Transforming Growth Factor-beta 1 (TGF-β1) is widely recognized as a master regulator of fibrosis, activating intracellular SMAD2/SMAD3 signaling, which promotes fibroblast-to-myofibroblast transition and ECM protein production, including fibronectin [8,9]. This pathway is well-characterized in systemic fibrotic diseases and has also been implicated in IUAs. Salma et al. (2016) reported increased TGF-β1 and SMAD3 expression in both patient samples and rabbit models of IUAs, identifying SMAD3 inhibition as a potential therapeutic approach [10]. Similarly, Xue et al. (2015) found that TGF-β1 and CCN2 were upregulated in IUAs and associated with NF-kB activation, linking fibrotic and inflammatory responses [11]. However, molecular data on isthmocele-associated fibrosis are limited. Histopathological studies have shown increased fibrosis, poor vascularization, and persistent inflammation in cesarean scar niches [7,12], suggesting a distinct and possibly more active fibrotic process. Younesi et al. (2024) highlight the role of sustained TGF-β1 signaling and myofibroblast persistence in chronic fibrotic disorders, which may be applicable to isthmocele [13].

Importantly, although classification systems (such as the American Fertility Score) help assess IUAs severity, the association between fibrosis-related gene expression and clinical stage remains unclear, with studies reporting inconsistent correlations [10,14]. Understanding the differential expression of key fibrotic markers in IUAs and isthmocele may aid in identifying biomarkers for diagnosis, prognosis, and targeted therapy.

The present study investigates for the first time the correlation between gene expression profiles of *TGF-β1*, *SMAD2*, *SMAD3*, and *fibronectin* in endometrial tissues collected from patients with IUAs and isthmocele compared with healthy patients. The main objective was to characterize the molecular signatures associated with these conditions and to explore the potential relevance of these fibrosis-related markers in differentiating IUAs from isthmocele, with particular emphasis on underlying fibrotic remodeling processes.

## 2. Materials and Methods

### 2.1. Study Design and Patient Selection

The prospective study included 55 patients with infertility, admitted to “Elena Doamna” Clinical Hospital of Obstetrics and Gynecology and “Cuza Voda” Clinical Hospital of Obstetrics and Gynecology in Iasi, Romania, between 2020 and 2024.

The study included women aged 20–45 years who were diagnosed with infertility, defined as failure to conceive after 12 months of unprotected intercourse. Eligible participants underwent diagnostic evaluation using hysteroscopy or transvaginal ultrasound to identify intrauterine pathology. Based on imaging and/or hysteroscopic findings, patients were assigned to one of three groups according to the presence and type of intrauterine pathology: IUAs group (n = 30 patients, 54.5%), isthmocele group (n = 9 patients, 16.4%), and control group (n = 16 patients, 29.1%). Patient selection was performed without discrimination based on gender, ethnicity, or religion.

All patients, including controls, underwent hysteroscopic evaluation and endometrial sampling during the proliferative phase of the menstrual cycle, in order to minimize variability related to cycle-dependent endometrial gene expression. Group allocation was based on consistent hysteroscopic and/or imaging criteria.

The control group consisted of women aged 20–45 undergoing routine gynecological evaluation, with normal hysteroscopic findings, no clinical symptoms suggestive of gynecological pathology, and no history of cesarean section. This selection strategy was adopted to minimize confounding factors related to uterine scarring, hormonal influences, and endometrial cycle phase, thereby allowing an appropriate comparison with the pathological groups.

Inclusion criteria required the availability of adequate endometrial tissue for RNA and histopathological analyses, the presence of regular menstrual cycles or cycle abnormalities related to uterine pathology, and written informed consent for participation and data use. Exclusion criteria included active pelvic infection, a history of gynecological malignancy or premalignant endometrial conditions, recent use of hormonal therapy (within 3 months), recent uterine surgery (within 6 weeks), known systemic condition affecting fertility (uncontrolled diabetes, thyroid disorders, or autoimmune diseases), incomplete clinical data and/or insufficient tissue quality for RNA analysis.

### 2.2. Ethical Approval

Ethical approval for this study was obtained in a stepwise manner, in accordance with the progressive design of the research and the institutional requirements of the participating centers. Initial patient recruitment and endometrial sample collection were approved by the Ethics Committee of the “Cuza Voda” Clinical Hospital of Obstetrics and Gynecology, Iasi, Romania (Approval No. 13881/15 December 2020). Following the extension of patient recruitment to the “Elena Doamna” Clinical Hospital of Obstetrics and Gynecology, Iasi, Romania, additional ethical approval was obtained (Approval No. 389/13 January 2023). Subsequently, ethical approval was granted by the Ethics Committee of “Grigore T. Popa” University of Medicine and Pharmacy Iasi, Romania, to authorize centralized molecular analyses and data processing (Approval No. 402/15 February 2024).

All research-related procedures, including RNA extraction, real-time quantitative PCR, and data analysis, were performed only after the corresponding ethical approvals had been obtained. All procedures were conducted in accordance with the ethical standards of the institutional and national research committees and with the 1964 Declaration of Helsinki and its later amendments. Written informed consent was obtained from all participants prior to inclusion in the study.

### 2.3. Diagnostic Procedures

Infertility was defined according to the guidelines of the American Society of Reproductive Medicine (ASRM) as the inability to conceive after 12 months of unprotected intercourse, or after 6 months in women aged 35 or older, including cases requiring medical intervention to achieve pregnancy [15]. Intrauterine adhesions were diagnosed using diagnostic hysteroscopy based on the AFS classification [16] and the isthmocele was confirmed via transvaginal ultrasound and hysteroscopy [17].

### 2.4. Sample Collection and Gene Expression Analysis

Endometrial tissue specimens were obtained during hysteroscopy using biopsy and preserved in DNA/RNA Shield (Zymo Research, Irvine, CA, USA) for subsequent RNA analysis. Samples were stored at −80 °C until further processing.

Total RNA was extracted from tissue samples previously preserved in DNA/RNA Shield by first thawing and transferring them to new sterile tubes, followed by homogenization in 1 mL of TRIzol^®^ reagent (Invitrogen, Thermo Fisher Scientific, Waltham, MA, USA), using a D1000 handheld homogenizer (Benchmark Scientific, Sayreville, NJ, USA). The homogenates were centrifuged at 12,000× *g* for 5 min at 4 °C to eliminate debris, and the resulting supernatants were transferred to fresh tubes. RNA isolation was performed according to the manufacturer’s protocol for TRIzol^®^ reagent.

Reverse-transcription of RNA was performed using the High-Capacity cDNA Reverse Transcription Kit on a Veriti PCR system (both from Applied Biosystems, Thermo Fisher Scientific, Waltham, MA, USA). Real-time quantitative PCR was performed using SyBr Select Real-Time PCR Master Mix (Applied Biosystems, Thermo Fisher Scientific, Waltham, MA, USA) and custom primers (Biolegio B.V., Nijmegen, The Netherlands) on a QuantStudio 12K Flex Real-Time PCR System (Applied Biosystems, Thermo Fisher Scientific, Waltham, MA, USA), according to the manufacturer’s protocols.

The custom primers used are presented in Table 1: SMAD family member 2 (*SMAD2*), SMAD family member 3 (*SMAD3*), transforming growth factor-beta 1 (*TGF-β1*), fibronectin 1 (*FN1*), and 18S ribosomal RNA (*18S*) as a reference gene. Amplifications were performed on a QuantStudio^TM^ 12K Flex Real-Time PCR System (Applied Biosystems, Thermo Fisher Scientific, Waltham, MA, USA) using 96-well plates, with all reactions run in technical duplicates. The *18S* ribosomal RNA gene was used as an endogenous reference control. Data were analyzed using QuantStudio^TM^ 12K Flex Software v1.2, with automatic baseline and threshold settings. Relative gene expression levels were calculated using the 2^−ΔCq^ method [18].

### 2.5. Statistical Analysis

Statistical analysis was conducted using the Statistical Package for the Social Sciences (SPSS, version 18.0; SPSS Inc., Chicago, IL, USA). Patient data were first compiled and structured for analysis. Subsequently, statistical comparisons were performed to extract supplementary parameters and insights. Continuous variables were expressed as mean ± standard deviation (SD) or median, with dispersion assessed using standard error and confidence intervals. Group comparisons were conducted using Student’s *t*-test, ANOVA or Bonferroni post hoc test, depending on data distribution and variable type. Categorical variables were analyzed using the Chi-square (χ^2^) test. Model performance was assessed using Receiver Operating Characteristic (ROC) curves and Area Under the Curve (AUC). A *p*-value < 0.05 was considered statistically significant [19,20]. Given the limited sample size and the hypothesis-generating nature of the study, all the statistical analyses were conducted within an exploratory framework and should be interpreted accordingly.

## 3. Results

### 3.1. Baseline Demographic Profile Among Participants

**Figure 1:**
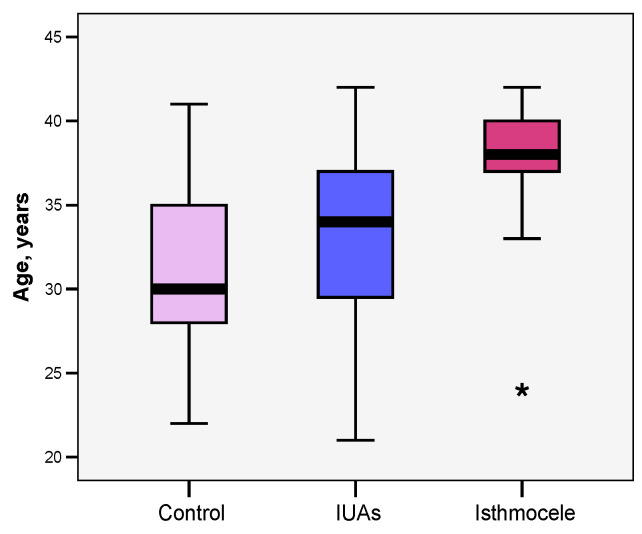
Comparison of mean age across study groups. IUAs—intrauterine adhesions. *—outlier value.

The age of the patients included in the study ranged from 21 to 42 years, with a mean value close to the median (32.67 vs. 33 years). The Skewness test result (−0.133) suggested that significance tests for continuous variables are applicable. The Bonferroni post hoc test highlights that the mean age was significantly higher in patients diagnosed with isthmocele (36.78 years) compared to the control group (31.13 years; *p* = 0.027). Although not statistically significant, a comparable mean age was observed in the group with IUAs (32.27 years; *p* = 0.160) (Figure 1).

### 3.2. Diagnostic Characteristics

Among all participants, IUAs were identified in 30 cases (54.5%), with 76.7% of these participants presenting with secondary infertility. Isthmocele was diagnosed in 9 patients (16.4%), all of whom had a history of at least one prior caesarean section and were affected by secondary infertility. Medical history revealed that pelvic inflammatory disease was significantly more prevalent among patients with IUAs (34.1%) compared to those with isthmocele (0%, *p* = 0.040). In contrast, other obstetric and gynecological factors—including first- and second-trimester abortions, post-abortion or postpartum curettage, biopsy curettage, myomectomy, hysteroscopy, uterine artery embolization, and congenital uterine malformations—did not differ significantly between the two groups (all *p* > 0.05) (Table 2).

### 3.3. Gene Expression Profiles of Endometrial Fibrosis Markers

#### 3.3.1. *TGF-β1* Expression

**Figure 2:**
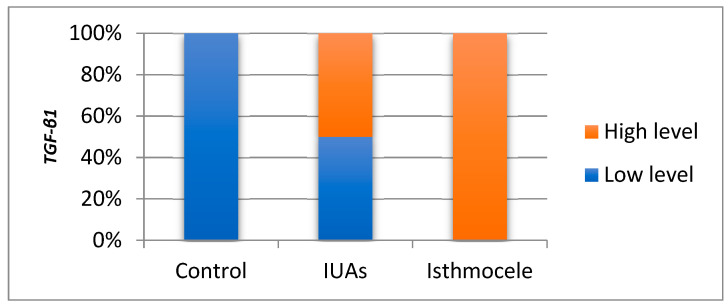
Comparison of *TGF-β1* expression level across the study group.

In the IUAs group, *TGF-β1* ranged from 0.014 to 0.976, with a mean and median of 0.421 and 0.449, respectively. Skewness test (0.361) indicated a homogeneous distribution suitable for parametric analysis. In the isthmocele group, values ranged from 0.458 to 5.101 (coefficient of variation, CV = 2.254), with a mean of 1.152 and a median of 1.121. Skewness test of 1.527 confirmed the applicability of statistical testing. The Bonferroni post hoc test reveals that the mean *TGF-β1* level was significantly higher than the IUAs group (1.852 vs. 0.421; *p* = 0.001). In the control group, levels ranged from 0.005 to 0.042, with a mean of 0.014 and a median of 0.013. The Skewness test (1.918) supported statistical testing. The Bonferroni post hoc test reveals that the mean level was significantly lower than in the isthmocele group (0.014 vs. 1.852; *p* = 0.001), but not significantly different from the IUAs group (*p* = 0.92). Because parametric tests may be affected by skewed gene expression data, non-parametric tests were applied. Using this approach, 50% of *TGF-β1* values in the IUAs groups and all values in the isthmocele group were above the median, with a statistically significant difference (*p* = 0.001) (Figure 2).

#### 3.3.2. *SMAD2* Expression

In the IUAs group, *SMAD2* expression ranged from 0.054 to 2.302, with a mean value of 0.530, closely approximating the median of 0.417. The Skewness value of 1.922 indicated an approximately normal distribution, supporting the use of a parametric statistical test. In the isthmocele group, *SMAD2* expression demonstrated high variability (CV = 17.108), with values ranging from 0.424 to 13.527. The mean expression level (2.892) was substantially higher than the median (1.324). Despite this dispersion, the Skewness value of 1.640 suggested a sufficiently normal distribution, justifying the application of a statistical significance test. In the control group, *SMAD2* levels ranged from 0.019 to 0.201, with a mean of 0.044 and a median of 0.035. The Skewness value of 1.652 confirmed an adequate distribution for the use of parametric tests.

The Bonferroni post hoc statistically significant difference was observed between the isthmocele and IUAs groups, with higher *SMAD2* levels in the isthmocele group (2.892 vs. 0.530; *p* = 0.001). *SMAD2* expression in the control group was significantly lower than in the isthmocele group (0.044 vs. 2.892; *p* = 0.001), but the difference between the control and IUAs group was not statistically significant (0.044 vs. 0.530; *p* = 0.615).

**Figure 3:**
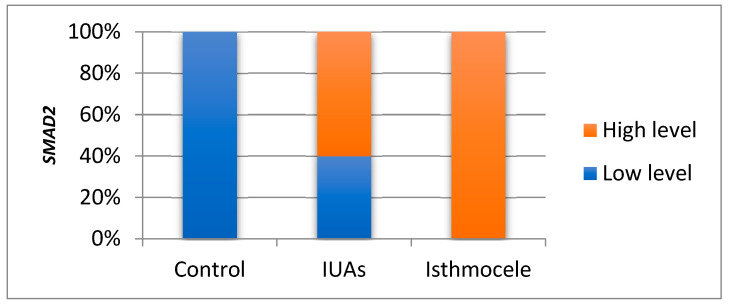
*SMAD2* levels in IUAs, isthmocele, and control groups; IUAs = intrauterine adhesions.

Using a non-parametric approach, 60% of the *SMAD2* values in the IUAs group exceeded the median value compared with 100% in the isthmocele group, with the difference remaining statistically significant (*p* = 0.001) (Figure 3).

#### 3.3.3. *SMAD3* Expression

In the IUAs group, *SMAD3* values ranged from 0.011 to 0.649, with a mean value close to the median (0.258 vs. 0.220). The result of the Skewness test was 0.468, indicating a homogeneous distribution, which allows for the application of statistical significance tests. In the isthmocele group, *SMAD3* levels varied widely (CV = 6.366) from 0.459 to 7.406, with the mean notably higher than the median (2.435 vs. 1.336). Despite this dispersion, the Skewness test result of 1.431 confirms a normal distribution, validating the use of parametric statistical tests. In the control group, *SMAD3* ranged from 0.002 to 0.021, with a mean value close to the median (0.005 vs. 0.004). The Skewness value of 1.959 also supports the use of statistical significance tests for continuous variables.

**Figure 4:**
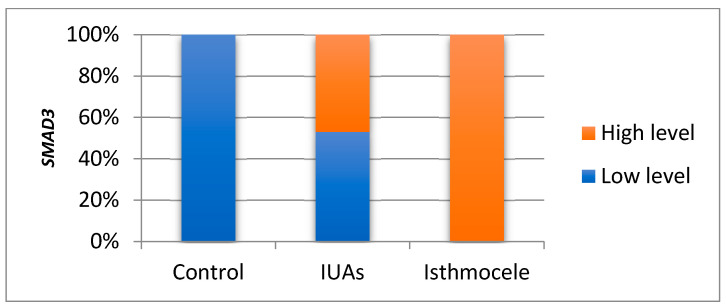
Comparison of *SMAD3* levels between the study groups.

The Bonferroni post hoc test highlights that the mean *SMAD3* level in the isthmocele group was significantly higher than that in the IUAs group (2.435 vs. 0.258; *p* = 0.001). The mean *SMAD3* level in the control group was significantly lower compared to the isthmocele group (0.005 vs. 2.435; *p* = 0.001), but the difference was not statistically significant when compared to the IUAs group (0.005 vs. 0.258; *p* = 0.696). Consistent with the non-parametric sensitivity analysis, 47% of the *SMAD3* values in the IUAs group exceeded the median, compared with 100% in the isthmocele group (*p* = 0.001) (Figure 4).

#### 3.3.4. *Fibronectin* Expression

**Figure 5:**
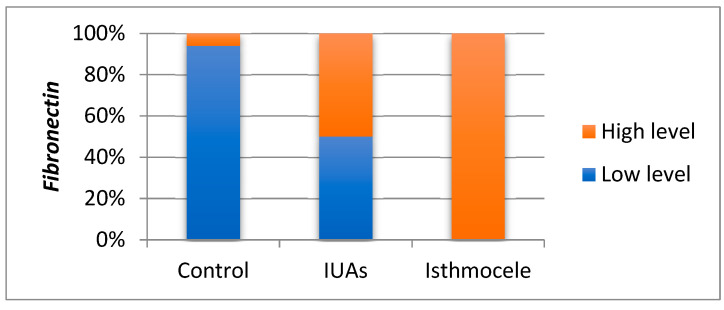
Comparisons of *fibronectin* mean values between groups.

In the IUAs group, *fibronectin* levels ranged from 0.007 to 0.571, with a mean of 0.094 and a median of 0.040. Skewness test (1.405) indicated a distribution suitable for parametric testing. In the isthmocele group, levels ranged from 0.084 to 0.764, with a mean of 0.348 and a median of 0.394. The skewness value of 0.338 confirmed data normality. The Bonferroni post hoc test highlights that the mean *fibronectin* level was significantly higher in the isthmocele group than in the IUAs group (0.348 vs. 0.094; *p* = 0.001). In the control group, levels ranged from 0.002 to 0.094, with a mean of 0.014 and a median of 0.008. Skewness test (1.432) supported the use of statistical tests for continuous variables. Also, the mean level was significantly lower than in the isthmocel group (0.014 vs. 0.348; *p* = 0.001), but not significantly different from the IUAs group (*p* = 0.263). Consistent with the non-parametric sensitivity analysis, 50% of the *fibronectin* values in the IUAs group and all values in the isthmocele group exceeded the median, with the difference remaining statistically significant (*p* = 0.001) (Figure 5).

### 3.4. Correlation and Predictive Utility of Endometrial Fibrosis—Associated Signaling Markers

Significant correlations were identified across the study population between *SMAD2* and *SMAD3* (r = 0.892; *p* = 0.001), *SMAD2* and *TGF-β1* (r = 0.697; *p* = 0.001), and *SMAD3* and *TGF-β1* (r = 0.910; *p* = 0.001). *TGF-β1* also correlated moderately with *fibronectin* (r = 0.384; *p* = 0.004), while *SMAD3* showed a weaker association (r = 0.268; *p* = 0.042). In subgroup analysis, no significant correlations were observed in the IUAs group (*p* > 0.05). However, in the isthmocele group, *SMAD2* and *SMAD3* were strongly correlated (r = 0.880; *p* = 0.002), as were *SMAD3* and *TGF-β1* (r = 0.880; *p* = 0.002).

**Figure 6:**
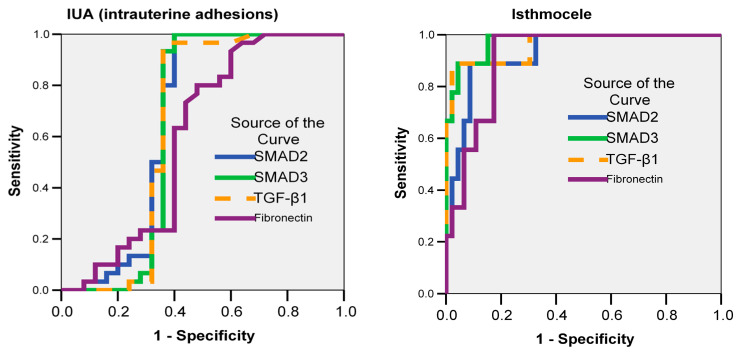
Receiver Operating Characteristic (ROC) analysis. Cellular signaling markers (endometrial fibrosis) as predictors of fibrosis and adhesion type.

ROC curve analysis (Figure 6) identified *SMAD2* as showing modest discriminatory ability for IUAs (AUC = 0.672; *p* = 0.029), with an optimal cut-off value of 0.29, while *SMAD3*, *TGF-β1*, and *fibronectin* did not reach statistical significance (*p* > 0.05), despite AUCs > 0.600. In contrast, all markers showed high discriminatory performance for isthmocele: *SMAD3* (AUC = 0.976; *p* = 0.001), *TGF-β1* (AUC = 0.961; *p* = 0.001), *fibronectin* (AUC = 0.913; *p* = 0.001), and *SMAD2* (AUC = 0.928; *p* = 0.001) with optimal cut-offs: 0.48 for *SMAD3*, 0.45 for *TGF-β1*, 0.87 for *fibronectin* and 0.41 for *SMAD2*. These findings highlight *SMAD3* and *TGF-β1* as highly reliable indicators of fibrotic signaling associated with isthmocele. However, given the small number of isthmocele cases, the results should be considered exploratory and require confirmation in larger cohorts.

### 3.5. Analysis of Fibrotic Markers’ Expression Across AFS Stages for IUAs Cases

Table 3 presents the number of values above the median for fibrosis-related cellular signaling markers (*TGF-β1*, *SMAD2*, *SMAD3*, and *fibronectin*) across different stages of IUAs, as classified by the AFS scoring system [16]. Stage I included 14 patients, and stage II and III were combined into a single group (n = 16), reflecting a moderate-to-severe disease category, due to the low number of patients in stage III (n = 3). Statistical comparisons were performed using the Chi-square or Fisher’s Exact Test, as appropriate, and the corresponding *p*-values are reported in the final column.

Fisher’s exact test showed no statistically significant differences between AFS stage I and stage II/III for *TGF-β1*, *SMAD2*, *SMAD3*, and *fibronectin* (all *p* > 0.05). Given the limited sample size, particularly in higher stages, these findings should be interpreted as exploratory, and no definitive conclusions can be drawn regarding stage-specific associations.

## 4. Discussion

In this study, real-time quantitative PCR was employed to assess the expression profiles and diagnostic relevance of key endometrial fibrosis-associated signaling markers (*TGF-β1*, *SMAD2*, *SMAD3*, and *fibronectin*) in endometrial tissue samples obtained from patients with IUAs and isthmocele, in comparison to healthy controls. Our findings reveal distinct molecular signatures underlying these two fibrotic gynecological pathologies, with particularly elevated markers’ expression in isthmocele, suggesting a more active and coordinated fibrotic signaling profile.

Accordingly, these biomarkers should be regarded primarily as indicators of underlying pathophysiological processes involved in fibrotic remodeling, rather than as clinically applicable diagnostic tools at this stage. Moreover, diagnostic performance metrics may be overestimated due to limited sample size and should, therefore, be interpreted within an exploratory framework.

### 4.1. Age and Clinical Associations

The observation that patients with isthmocele were significantly older than controls (mean age: 36.78 vs. 31.13 years; *p* = 0.027) is consistent with existing evidence implicating advanced maternal age as a risk factor for caesarean-related defects and secondary infertility. A systematic review has reported an increased risk of caesarean delivery with advancing maternal age (RR 1.4–2.8), while cesarean rates reach up to 43% in women aged ≥40 years, compared with 11.6% in women under 25 years [21]. In line with these data, all patients with isthmocele in our cohort had a history of caesarean delivery and secondary infertility. A meta-analysis further supports this association, reporting secondary infertility in approximately 43% of women with symptomatic isthmocele, reinforcing a causal link between surgical trauma and localized endometrial fibrosis [6].

In contrast, patients with IUAs exhibited an age distribution comparable to that of controls, with no statistically significant difference (*p* = 0.077). This likely reflects the broader and more heterogeneous etiological spectrum of IUAs, which includes infection, curettage, and post-abortive complications—factors that are not necessarily age-dependent.

### 4.2. Fibrosis Markers’ Expression and Diagnostic Relevance

The most pronounced molecular alterations were observed in the isthmocele group, where all four investigated markers were significantly upregulated compared with both IUAs and controls. *SMAD2* expression was markedly elevated in isthmocele (mean = 2.892) relative to IUAs (mean = 0.530; *p* = 0.001) and control (mean = 0.044; *p* = 0.001), with similar expression patterns observed for *SMAD3* (2.435 vs. 0.258 vs. 0.005, respectively), *TGF-β1* (1.852 vs. 0.421 vs. 0.014), and *fibronectin* (0.348 vs. 0.094 vs. 0.014), all with *p* < 0.001. The strong intercorrelations among these markers further support the presence of a coherent and robust profibrotic signaling cascade.

These findings are consistent with the well-established role of TGF-β1/SMAD signaling as a central driver of fibrosis. Wynn (2008) described TGF-β1 as the principal fibrogenic cytokine, orchestrating ECM deposition and myofibroblast activation through SMAD-dependent pathways [8]. The concurrent upregulation of *TGF-β1*, *SMAD2*, and *SMAD3* in isthmocele observed in our study provides molecular confirmation of this framework in post-cesarean uterine fibrotic remodeling. Moreover, the increased expression of *fibronectin*—a key ECM glycoprotein—supports the presence of active-matrix remodeling and myofibroblast-mediated scar formation, processes closely linked to epithelial-and endothelial-to-mesenchymal transition (EMT/EndMT) [8].

Recent advances further emphasize the pivotal role of SMAD3 as a transcriptional hub in fibrotic disease through its regulation of non-coding RNAs (ncRNAs). Gu et al. (2024) demonstrated that SMAD3-dependent microRNAs (miRNAs) and long ncRNAs amplify and sustain profibrotic gene expression, promoting ECM accumulation and myofibroblast persistence across multiple organ systems [22]. In parallel, Meng et al. (2016) characterized TGF-β as the “master regulator” of fibrosis, highlighting the critical contribution of RNA-level regulation—including epigenetic mechanisms—to SMAD-mediated fibrotic programs [23]. Similarly, reviews focusing on IUAs have underscored the role of miRNAs-mediated regulation of TGF-β1 signaling in disease initiation and recurrence [24]. Collectively, these findings support the interpretation that altered gene expression regulation at the RNA level contributes to the pronounced activation of the *TGF-β1/SMAD2/SMAD3* axis observed in isthmocele, and suggest that *SMAD3* may represent both a biomarker of fibrotic burden and a promising therapeutic target amenable to ncRNA-based intervention strategies.

In contrast, although fibrosis-associated markers were elevated in IUAs, only *SMAD2* demonstrated limited discriminative performance (AUC = 0.6672; *p* = 0.029), and no significant correlations among markers were detected. This pattern suggests a less uniform, stage-dependent, or context-specific fibrotic response, potentially influenced by heterogeneous etiologies such as mechanical trauma or inflammation, rather than a dominant SMAD-centered fibrotic program. These observations are in agreement with previous studies reporting upregulated TGF-β1 and SMAD3 expression in IUAs, alongside downregulation of the inhibitory SMAD7, and demonstrating attenuation of fibrosis following pharmacological SMAD3 inhibition [10].

Beyond canonical TGF-β1/SMAD signaling, fibrotic remodeling is increasingly recognized as the result of interconnected intracellular signaling networks. Guo et al. (2017) demonstrated that TGF-β1 interacts with the GSK-3/β-catenin axis to regulate fibroblast activation, myofibroblast differentiation, and ECM accumulation in myocardial fibrosis [25]. Such signaling cross-talk suggests that SMAD-dependent pathways operate within broader regulatory frameworks, which may contribute to the robust and coordinated fibrotic profile observed in isthmocele.

Furthermore, previous studies investigating IUAs provide additional context for the more heterogeneous fibrotic profile observed in this group. Xue et al. (2015) employed qRT-PCR alongside immunohistochemistry and Western blotting and demonstrated significant overexpression of both TGF-β1 and CCN2 in adhesion tissue, which was concomitantly associated with activation of the NF-kB pathway [11]. These findings underscore the close interplay between fibrogenic and inflammatory signaling in IUAs and suggest that inflammatory cues may substantially modulate fibrotic gene expression.

In partial contrast to these reports, our data reveal that although IUAs exhibited elevated expression of *SMAD2* with modest discriminatory ability (AUC = 0.6672; *p* = 0.029), no significant upregulation of *SMAD3*, *TGF-β1*, or *fibronectin* was detected. This discrepancy may reflect the biological heterogeneity of IUAs, differences in disease stage, or variability in the underlying etiological drivers, including mechanical trauma, infection, and chronic inflammation. Together, these observations suggest that fibrotic remodeling in IUAs may not uniformly depend on sustained activation of the potentially transient signaling events.

Complementary evidence from experimental models further supports this notion. In murine models of IUAs, real-time PCR and immunohistochemistry analysis revealed significant upregulation of fibrosis-related genes, including TGF-β1, CTGF, and collagens I and III, as well as stem cell markers such as CD146, CD140b, in adhesion tissue compared with normal endometrium (*p* < 0.05) [26]. While these findings confirm the presence of a fibrosis-driven endometrial microenvironment, such studies did not stratify adhesion subtypes or directly compare IUAs with isthmocele, limiting their ability to capture disease-specific molecular signatures.

In contrast, ROC curve analysis in the present study demonstrated high discriminatory performance of all four markers in isthmocele, with AUC values exceeding 0.91. *SMAD3* (AUC = 0.976) and *TGF-β1* (AUC = 0.961) emerged as particularly powerful discriminators, reinforcing the concept that *SMAD3* represents a central mediator of fibrotic burden across organ systems [9]. Although these markers were also elevated in IUAs, only *SMAD2* achieved statistical significance for predictive utility (AUC = 0.6672; *p* = 0.029), further supporting a more heterogeneous or less advanced fibrotic process in this condition.

Recent advances have further highlighted the importance of ncRNAs as critical downstream regulators of TGF-β1/SMAD-mediated fibrosis. A comprehensive review by Xu et al. (2021) demonstrated that multiple microRNAs and long ncRNAs modulate SMAD2/3 activity, thereby fine-tuning fibrogenic gene expression, myofibroblast persistence, and ECM deposition across diverse fibrotic diseases [27]. Within this mechanistic framework, the pronounced and coordinated upregulation of *SMAD3* observed in isthmocele may facilitate sustained profibrotic signaling through ncRNA-dependent regulatory loops, contributing to the more uniform and active fibrotic phenotype identified in this pathology.

### 4.3. Distinct Fibrotic Profiles: Isthmocele vs. IUAs

Although both isthmocele and IUAs represent fibrotic uterine conditions, the molecular profiles identified in this study indicate distinct pathophysiological mechanisms. Isthmocele is characterized by a coordinated upregulation of *TGF-β1*, *SMAD2*, *SMAD3*, and *fibronectin* with strong inter-correlations (*r* > 0.88, *p* < 0.01), indicative of an active and sustained fibrotic response. These findings are consistent with previous reports describing isthmocele as a niche of poor vascularization, chronic inflammation, and progressive fibrosis at the site of myometrial discontinuity following cesarean section [7].

In contrast, the absence of significant marker correlations in IUAs suggests greater molecular heterogeneity, potentially reflecting variable contributions from mechanical injury, inflammation, and surgical technique [5]. Sustained activation of the TGF-β1/SMAD2/3 axis has been shown to promote EMT and myofibroblast differentiation, thereby perpetuating fibrosis through impaired resolution of wound healing [28]. The coordinated marker overexpression observed in isthmocele is therefore consistent with a more EMT-driven and self-sustaining fibrotic program, whereas the heterogeneous expression patterns in IUAs may reflect partial or transient pathway activation. Notably, the significant upregulation of fibronectin—a key ECM glycoprotein involved in tissue repair and remodeling—further supports the presence of active matrix deposition and scar formation in this condition [7,12,29].

Emerging transcriptomic evidence further supports this interpretation. Recent reviews synthesizing bulk and single-cell RNA analyses demonstrate that fibrotic signaling in IUAs varies across cell populations and injury contexts, resulting in non-uniform gene expression profiles [30]. Such transcriptional heterogeneity may underlie the variable diagnostic performance of fibrosis-related markers observed in IUAs compared with isthmocele.

The recent work by Younesi et al. (2024) on fibroblast and myofibroblast activation in tissue repair and fibrosis [13] offers a valuable lens to interpret fibrotic endometrial conditions such as isthmocele and IUAs. In line with this model, our data demonstrate that isthmocele exhibits a coordinated upregulation of *TGF-β1*, *SMAD2/3*, and *fibronectin*, indicative of an active and sustained fibrotic response. This pattern is consistent with prolonged TGF-β1 signaling driving myofibroblast differentiation and persistence, particularly within poorly vascularized and chronically inflamed microenvironments such as the cesarean scar niche characteristic of isthmocele [13]. In contrast, these comparatively weaker and less coordinated marker expressions observed in IUAs suggest a more heterogeneous fibrotic process. Collectively, these findings indicate that although both conditions are fibrotic in nature, they are governed by distinct molecular programs—a distinction that may have important implications for the development of targeted and condition-specific antifibrotic therapeutic strategies.

### 4.4. Markers’ Gene Expressions Across AFS Stages in IUAs

Although an overall trend toward increased expression of fibrotic markers with advancing AFS stage was observed in patients with IUAs, none of these differences reached statistical significance (*p* > 0.05). For example, *SMAD2* expression was highest in stage III (mean = 0.865) and lowest in stage I (mean = 0.319), approaching but not achieving statistical significance (*p* = 0.060). Comparable non-significant upward trends were noted for *SMAD3* and *TGF-β1*. In contrast, fibronectin expression was unexpectedly lower in stage III adhesions, further underscoring the heterogeneity of fibrotic remodeling across disease stages.

These findings partially contrast with previous reports indicating increased TGF-β1 and SMAD signaling in moderate IUAs [10], as well as studies demonstrating significant correlations between IUA severity (grades I-III) and metalloproteinase activity, including ADAM-15 and ADAM-17 levels (*p* < 0.01) [14]. Such discrepancies may reflect differences in cohort size, disease chronicity, sampling strategies, or the intrinsic limitations of the AFS classification system in capturing molecular disease activity.

Collectively, these observations highlight substantial biological variability within IUAs and underscore the need for larger, well-characterized cohorts to determine whether molecular marker expression can reliably predict adhesion severity as defined by AFS staging [16]. Previous investigations have reported inconsistent associations between histological, molecular, and clinical severity metrics, potentially due to variability in tissue sampling and subjective scoring methodologies. Beyond TGF-β1/SMAD signaling, proteomic analyses have identified additional ECM-related proteins—including ADIPOQ, MYLK, CAMK2G—as contributors to IUA pathophysiology, suggesting the involvement of broader molecular networks [31].

Moreover, stem cell-associated fibrotic markers such as CTGF and collagens I and III have been shown to be elevated in both human and murine IUAs models, supporting a fibrosis-prone endometrial microenvironment [26]. Inflammatory factors may further modulate the extent and uniformity of fibrotic remodeling in IUAs. Notably, Liu et al. (2019) demonstrated that chronic endometritis significantly exacerbates endometrial fibrosis and negatively impacts reproductive outcomes in patients with moderate to severe IUAs, identifying inflammation as a critical co-factor influencing the activation and detectability of TGF-β1/SMAD-mediated fibrotic signaling [32]. Taken together, these findings emphasize the complexity of IUAs pathogenesis and support the expansion of both molecular panels and sample sizes in future studies to more accurately stratify disease severity and guide personalized therapeutic strategies.

Overall, these findings should be interpreted within the context of an exploratory analysis and in light of emerging evidence highlighting isthmocele as a clinically relevant post-cesarean condition amendable to minimally invasive repair [33], underscoring the need for larger, well-powered, and age-stratified studies to validate the observed associations and to clarify the role of fibrosis-related signaling pathways in uterine pathology [34].

## 5. Conclusions

This study provides novel molecular evidence that distinguishes isthmocele from IUAs in terms of fibrosis-related signaling. The significantly elevated and coordinated expression of *TGF-β1*, *SMAD2*, *SMAD3*, and *fibronectin* observed in isthmocele reflects activation of post-cesarean fibrotic remodeling pathways. However, several methodological and interpretative limitations—including the small sample size, heterogeneity of the study population, and the exploratory nature of the analyses—preclude definitive conclusions regarding clinical applicability or predictive performance. These findings should therefore be regarded as hypothesis-generating and indicative of underlying fibrotic processes. Further studies in larger, well-characterized cohorts are required to validate these findings, to clarify their biological significance, and to assess their potential relevance for prognostic stratification or therapeutic targeting in post-cesarean uterine pathology and IUAs.

## Figures and Tables

**Table 1:** Primer sequences and amplicon size used for gene expression analysis by real-time PCR.

Gene	Gene ID	Sequence 5′-3′	Amplicon Size (bp)
***18S***	NR_145820.1	F 5′ GGAGCCTGCGGCTTAATTTG 3′ R 5′CCACCCACGGAATCGAGAAA 3′	100
***TGF-β1***	NM_000660.7	F 5′ AGACGGATCTCTCTCCGACC 3′ R 5′ GGTGTCTCAGTATCCCACGG 3′	106
***SMAD2***	NM_001003652.4	F 5′ TACACCAAATACGATAGATCAGTGG 3′ R 5′ GGAGACGACCATCAAGAGACC 3′	83
***SMAD3***	NM_005902.4	F 5′ GGTTGGACTTTCCTTCCCG 3′ R 5′ AAGTGGCAGCAGAAGTTTGG 3′	74
***Fibronectin-1***	NM_212482.4	F 5′ CGGGACTCAATCCAAATGCC 3′ R 5′ CCAGGAACCCTGAACTAAGG 3′	148

**Table 2:** Medical history of patients with intrauterine adhesions and isthmocele.

Patient History	IUAs (n = 44)	Isthmocele (n = 9)	Chi-Square Test–*p*
**Obstetric history**			
Abortion			
First Trimester	29 (65.9%)	4 (44.4%)	0.230
Second Trimester	5 (11.4%)	-	0.293
No abortion	10 (22.7%)	5 (55.6%)	0.048
Postabortum D&C	25 (56.8%)	4 (44.4%)	0.501
Postpartum D&C	4 (9.1%)	0 (0.0%)	0.351
Caesarean section	0 (0.0%)	9 (100%)	0.001
**Gynaecological history**			
Biopsy curettage	4 (9.1%)	0 (0.0%)	0.351
Myomectomy	4 (9.1%)	2 (22.2%)	0.262
Hysteroscopy	9 (20.5%)	1 (11.1%)	0.518
Uterine artery embolization	1 (2.3%)	0 (0.0%)	0.651
**Pathophysiological disorders**			
Pelvic inflammatory disease	15 (34.1%)	0 (0.0%)	0.040
Congenital uterine malformations	6 (13.6%)	0 (0.0%)	0.244

IUAs—intrauterine adhesions; D&C—dilation and curettage.

**Table 3:** Mean expression of fibrosis-related cellular signaling markers according to the AFS scoring system for IUAs (F_ANOVA_ test).

Cellular Signaling Markers Associated with Fibrosis	AFS Score		
	Stage I (n = 14)	Stage II/III (n = 16)	Fisher’s Exact Test *p*
High values of *TGF-β1*	5 (35.7%)	10 (62.5%)	0.136
High values of *SMAD2*	6 (42.9%)	12 (75.0%)	0.078
High values of *SMAD3*	4 (28.6%)	10 (62.5%)	0.067
High values of *fibronectin*	6 (42.9%)	9 (56.3%)	0.358

## Data Availability

The data and original contributions supporting the findings of this study are fully documented within the article. Any further requests for information should be addressed to the corresponding author.
